# Association Between Child Welfare Facilities and Distribution of Doctors in Japan: An Observational Study

**DOI:** 10.7759/cureus.101264

**Published:** 2026-01-10

**Authors:** Hiromitsu Nagano, Soshi Takao

**Affiliations:** 1 Department of Epidemiology, Graduate School of Medicine, Dentistry and Pharmaceutical Sciences, Okayama University, Okayama, JPN

**Keywords:** childcare environment, child welfare, distribution of doctors, human resources, physician uneven distribution indicator

## Abstract

Introduction

Child welfare facilities (CWFs) may be crucial in determining the distribution of doctors. However, research investigating the relationship between the CWFs and the distribution of doctors in Japan is limited. Therefore, this study examined the relationship between the CWFs and the distribution of doctors across secondary medical areas in Japan.

Methods

The study considered secondary medical areas in the Kinki and Chugoku regions. The change in the number of CWFs was represented by four categorical variables (0 or fewer, 1-4, 5-9, and 10 or more). The distribution of doctors was defined as the adjusted number of doctors per 100,000 people and was represented by three categorical variables and a continuous variable. We used ordinal logistic and linear regression analyses to assess the association between the change in the number of CWFs and the adjusted number of doctors per 100,000 people, after adjusting for medical expense subsidies for infants, retail sales floor area per population, income per taxpayer, and hours of sunlight.

Results

Across 71 secondary medical areas in Japan, a statistically significant association was observed between the group with an increase of 10 or more CWFs and the adjusted number of doctors per 100,000 people (odds ratio: 5.1; 95% confidence interval: 1.02 to 25.8). Additionally, this group had an increased adjusted number of doctors per 100,000 people, which was more than 50 higher than that of the group with no increase in CWFs.

Conclusion

These findings suggest that there may be an association between increasing CWFs and the adjusted number of doctors per 100,000 people.

## Introduction

In Japan, various policies aimed at improving the distribution of doctors have been implemented since the 1970s. However, regional disparities in the distribution of doctors have not been sufficiently alleviated, up to the present time, and several studies have suggested that maldistribution has worsened [1-3]. In response to this situation, revisions to the “Act for the Partial Revision of the Medical Care Act and Medical Practitioners Act” in 2018 required each prefecture to formulate and implement plans for securing doctors based on regional healthcare needs. Furthermore, in 2024, with the launch of the Eighth Medical Care Plan, plans for securing doctors were updated in each prefecture, and new plans to address the distribution of doctors are currently being implemented.

Studies on the distribution of doctors in Japan have reported that factors such as the housing environment, the ability to maintain specialist certification, and the desire to belong to ikyoku (the clinical department of a medical school characterized by a professor at the top of the hierarchy) influence doctors’ workplace choice [4-6]. However, limited research has examined the relationship between child welfare facilities (CWFs) and the distribution of doctors. Tomizawa et al. conducted a survey on work-related and personal stress among members of the Japan Surgical Society who were raising children. The results showed that “seeking a temporary place to care for their child” ranked the highest among daily stressors for male and female surgeons. As the participants specifically belonged to the Japan Surgical Society, these results may not be generalizable to all doctors. Nevertheless, this study suggests that the CWFs are likely to be a significant factor in the lives of Japanese doctors [7].

Therefore, this study examined the relationship between the CWFs and the distribution of doctors across secondary medical areas in Japan, which are the regional units for medical care delivery.

## Materials and methods

**Figure 1:**
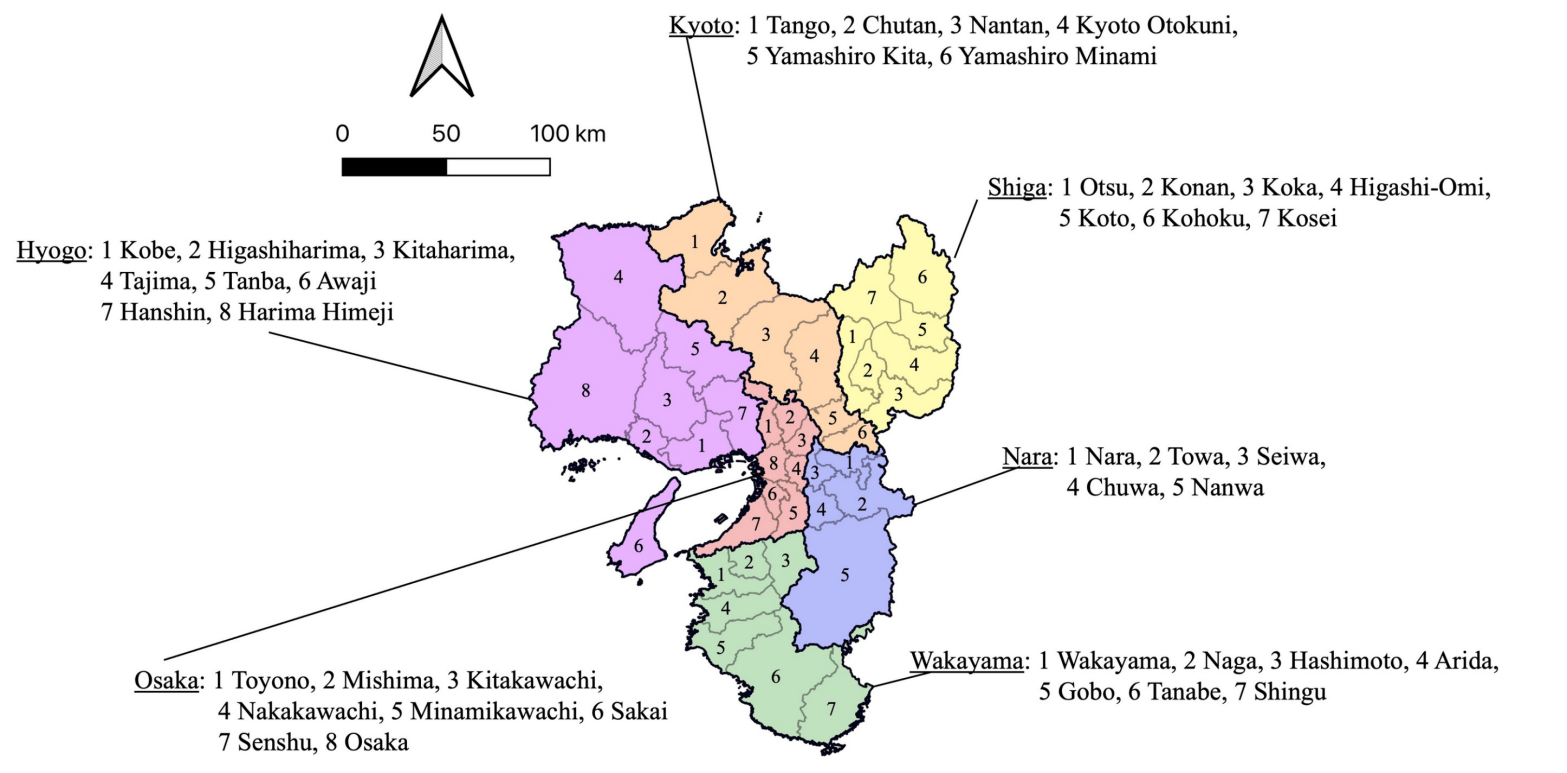
Secondary medical areas in the Kinki region. The Kinki region comprises six prefectures: Shiga, Kyoto, Osaka, Hyogo, Nara, and Wakayama. The number of secondary medical areas in the Kinki region was 41.

**Figure 2:**
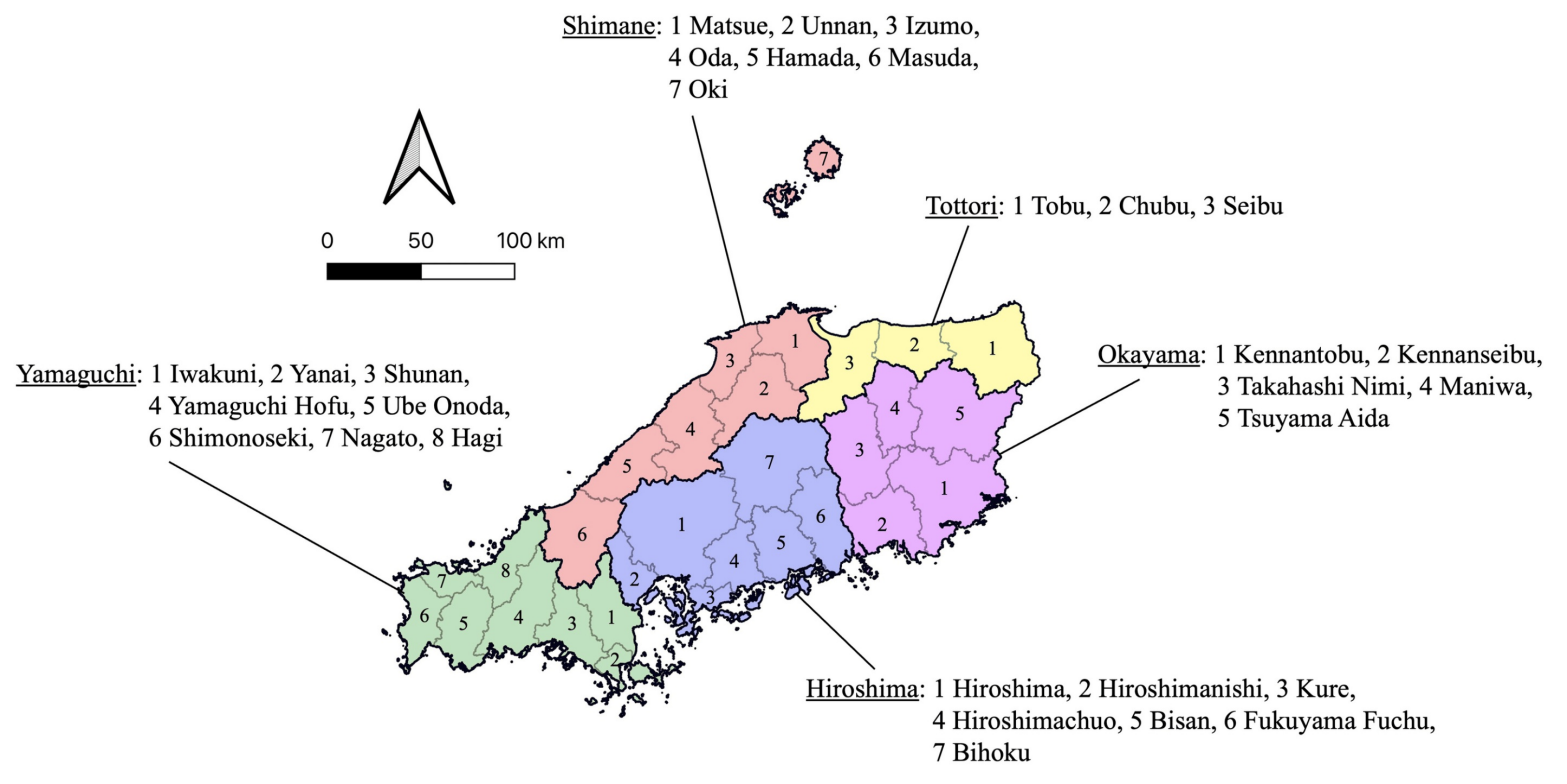
Secondary medical areas in the Chugoku region. The Chugoku region comprises five prefectures: Tottori, Shimane, Okayama, Hiroshima, and Yamaguchi. The number of secondary medical areas in the Chugoku region was 30.

The study subjects were areas for medical care delivery. A previous study has suggested that using a municipality as a unit of analysis is not necessarily appropriate when considering the distribution of doctors [8]. Following the methodology of Tanihara et al. [3], this study targeted secondary medical areas, which are regional units capable of providing general inpatient services, including emergency care, and comprise multiple municipalities. Primary and tertiary medical areas generally correspond to a single municipality and prefecture, respectively. In Japan, the number of doctors per capita is lower in the east and higher in the west. Therefore, including the entire country is not feasible. Instead, the study focused on the western part, and the Kinki and Chugoku regions were included. Figure 1 presents the secondary medical areas in the Kinki region. Figure 2 presents the secondary medical areas in the Chugoku region. The data were obtained from open data sources published by the relevant government ministries. As these were municipal data, they were aggregated as per their corresponding secondary medical areas.

We used data from different time periods to address the potential reverse causality in the relationship between the exposure (2014-2016) and the outcome (2022). During this period, there were no policy interventions that directly promoted increases in the CWFs, and plans for securing doctors were initiated only after 2018. Therefore, the exposure used in this study is unlikely to have been influenced by policy-driven initiatives based on expected future increases in doctor supply. Approval from an ethics committee was not required as this study used publicly available open data.

Exposure variable

The exposure variable was the change in the number of CWFs per secondary medical area. This change was calculated as the difference between 2014 and 2016. The data were obtained from the “Survey of Social Welfare Facilities” conducted by the Ministry of Health, Labor and Welfare (MHLW). These surveys outline the types of CWFs [9,10], with most facilities being nursery schools. The changes in the number of CWFs were divided into four groups (0 or fewer, 1-4, 5-9, and 10 or more) according to the quantiles.

Outcome variable

The outcome variable was the adjusted number of doctors per 100,000 people, which was calculated by the MHLW [11]. The figure was adjusted for medical demand (standardized rate of hospital visits) and supply (standardized number of doctors calculated from the average working hours by gender and age group). The adjusted number of doctors was divided into tertiles (Q1: the highest; Q3: the lowest).

Covariate

Indicators in the “Favorable Living Environment: Municipality Ranking 2024” published by Toyo Keizai Inc. were employed as covariates [12]. All the indicators are listed in Table 1. Because of multicollinearity concerns, only one indicator was selected from each domain: medical expense subsidies for infants (safety), retail sales floor area per capita (convenience), hours of sunlight (comfort), and income per taxpayer (affluence). Scores were calculated for each municipality and aggregated according to the secondary medical area to which each municipality belonged. An average score was assigned to each secondary medical area.

**Table 1:** Indicators used in the “Favorable Living Environment: Municipality Ranking 2024,” published by Toyo Keizai Inc. Source: [12] The boldfaced text highlights the indicators used in this study. *Because of data availability, the retail sales floor area was used instead of the floor space of large-scale retail stores.

Safety level	Number of hospitals and general clinics per capita
	Number of beds in long-term care, welfare, and health facilities per older adult
	Number of children aged 0-4 per female population aged 20-39
	Medical expenses subsidies for infants
	Number of recognized criminal offenses per capita
	Number of traffic accidents per capita
Convenience level	Retail sales per capita
	Floor space of large-scale retail stores per capita*
	Number of food and beverage retail establishments per unit of inhabitable land area
	Number of restaurants per capita
Comfort level	Inflow and outflow rate
	Water bill
	Sewage treatment coverage rate
	Climate (monthly average high and low temperatures, hours of sunlight, and maximum snow depth)
	Urban park area per capita in designated urban planning areas
Affluence level	Fiscal capacity index
	Corporate tax per capita
	Income per taxpayer
	Total floor area per dwelling
	Average land value

The medical expense subsidies for infants were quantified using the original scoring system. Details of the scoring components are provided in Appendix 1. In this study, the scores were based on the 2016 report titled “Implementation Status of Medical Expense Subsidies for Infants in Municipalities,” published by the MHLW [13]. The scores were assigned according to the patient’s age, eligibility for medical expense assistance for outpatient and hospitalization, the presence or absence of an income limit, and the presence or absence of partial out-of-pocket payment. The scores for each municipality were then aggregated within each secondary medical area and averaged to obtain a representative score for that area. The score was designed to reflect the sufficiency of municipal medical subsidies for infants and was not intended to indicate the relative importance of each component. The score ranged from 2 to 12, with a higher score indicating greater sufficiency. The hours of sunlight were based on data from 2010, obtained from the National Land Numerical Information provided by the Ministry of Land, Infrastructure, Transport and Tourism. The hours of sunlight have been reported to show relative stability on an annual scale, so the 2010 data were used as a proxy for the 2016 data [14]. The other indicators were calculated as follows:

$\text{Retail sales floor ares per population}\left[ m^{2} \right]=\text{Retail sales floor area} / \mathrm{Population}$

$\text{Income per taxpayer}\left[ 100,000 yen \right]= \left( \text{Total taxable income} / 100 \right)/\mathrm{Taxpayer}$

Statistical analysis

The overall characteristics and each group’s (Q1-Q3) characteristics for the adjusted number of doctors per 100,000 people were described using means and standard deviations for continuous variables and counts and percentages for categorical variables.

We conducted an ordinal logistic regression analysis to assess the association between changes in the number of CWFs and the adjusted number of doctors per 100,000 people. The results were shown for both crude analyses and analyses adjusted for medical expense subsidies for infants, retail sales floor area per population, income per taxpayer, and hours of sunlight. We estimated the crude and adjusted odds ratios and 95% confidence intervals (CIs) for a one-step increment in tertile groups (i.e., Q3 to Q2 or Q2 to Q1) association with categorized changes in the number of CWFs. The proportional assumption in the ordinal logistic regression analysis was assessed using the Brant test [15]. Furthermore, we conducted a linear regression analysis to assess the dose-response relationships. The crude and adjusted beta coefficients (the actual number of expected increases in doctors) and 95% CIs associated with the same categorized exposure were estimated. In addition, as a sensitive analysis, we conducted another linear regression analysis to estimate the actual expected number of doctors associated with an increase in CWFs and nursery schools. We used restricted cubic splines to examine the assumption of the linearity of the association between changes in the number of CWFs and the adjusted number of doctors per 100,000 people. No adjustment of multiple comparisons was applied because the analysis addressed the same primary hypothesis using different analytical approaches. A p-value of less than 0.05 was considered statistically significant. The statistical analyses were performed using Stata SE version 18 (StataCorp, College Station, TX, USA).

## Results

Table 2 presents the characteristics of the secondary medical areas in Kinki and Chugoku (n = 71). The Q1 group had a higher population, number of doctors, and income per taxpayer; more CWFs and nursery schools; and longer hours of sunlight. By contrast, the medical expense subsidies for infants were lower in this group. The Q1 group also had a higher proportion of increases of 10 or more CWFs (45.8%). Conversely, the Q2 group had a higher proportion of increases of five to nine CWFs (29.2%). The Q3 group observed a higher proportion of increases of zero or fewer in the number of CWFs (60.9%).

**Table 2:** Characteristics of secondary medical areas in the Kinki and Chugoku regions. SD: Standard deviation; Freq: Frequency

		Q1 (n = 24)			Q2 (n = 24)		Q3 (n = 23)	
		Mean	SD		Mean	SD	Mean	SD
Population in 2020 (/1,000 people)		681.7	680.7		356.4	321.7	126.0	107.4
Adjusted number of doctors per 100,000 people		308.4	48.4		218.7	10.1	175.5	21.8
Medical expenses subsidy for infants (points)		8.1	1.9		8.5	1.8	8.8	1.8
Retail sales floor area per population (m^2^)		1.1	0.2		1.1	0.2	1.2	0.3
Income per taxpayer (100,000 yen)		31.9	3.8		29.2	2.7	27.6	2.6
Hours of sunlight (hours)		1,757.9	188.2		1,804.5	160.0	1,814.3	160.6
Number of child welfare facilities								
2016		164.0	154.1		82.2	53.3	42.3	28.4
2014		141.2	127.5		76.4	47.9	41.6	27.5
Number of nursery schools								
2016		111.8	96.8		65.4	43.7	34.6	23.8
2014		104.3	91.0		63.3	41.1	34.4	23.2
Change in the number of child welfare facilities		Count	%		Count	%	Count	%
0 or fewer		3	12.5		6	25.0	14	60.9
1-4		5	20.8		6	25.0	6	26.1
5-9		5	20.8		7	29.2	2	8.7
10 or more		11	45.8		5	20.8	1	4.4
Local public entity		Freq	%		Freq	%	Freq	%
Prefectural capital location		9	7.6		2	1.9	0	0
Other cities		58	49.2		62	57.9	35	43.8
Town		37	31.4		43	40.2	40	50.0
Village		14	11.8		0	0	5	6.2

**Figure 3:**
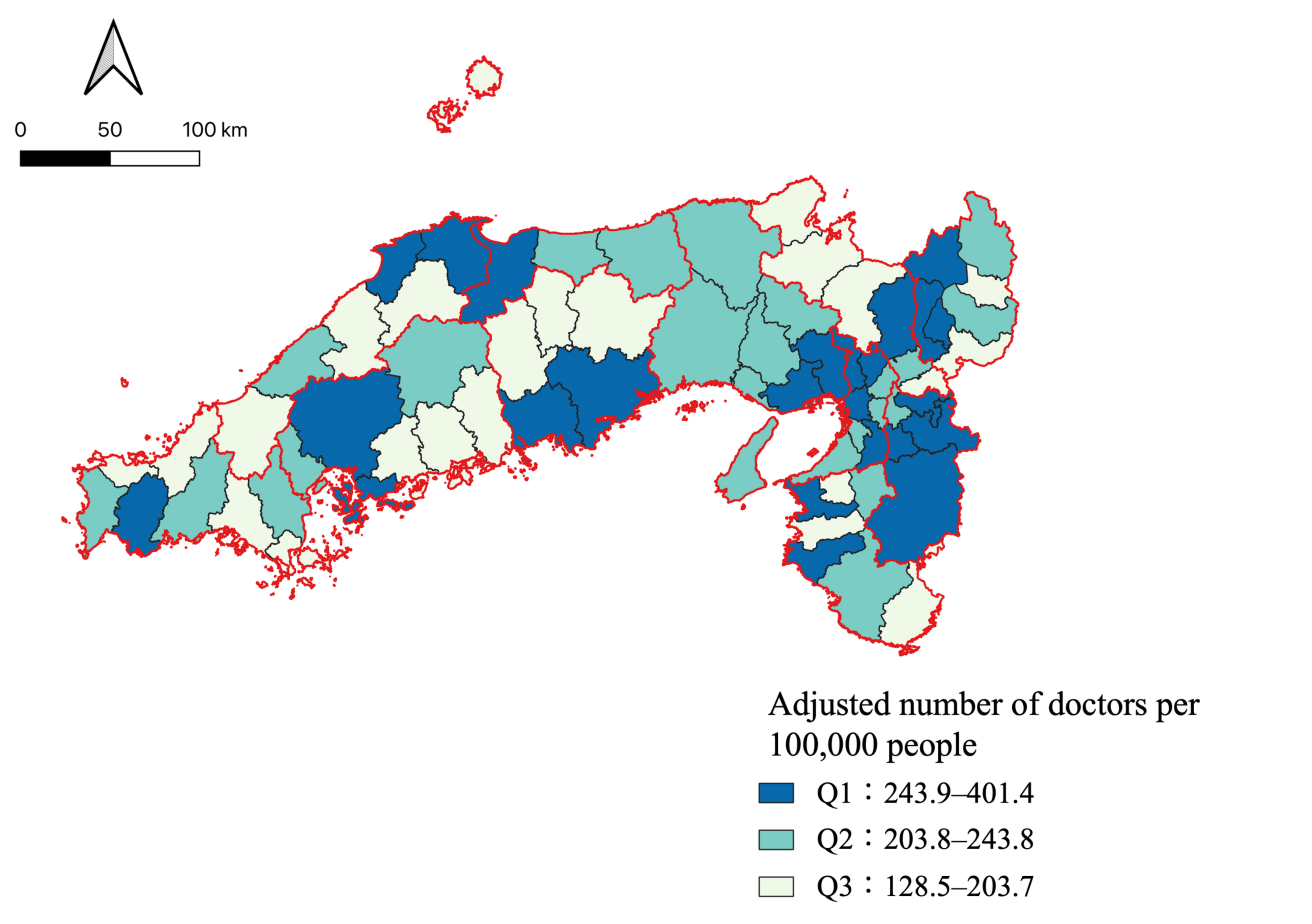
Adjusted number of doctors per 100,000 people for each secondary medical area.

Figure 3 illustrates the distribution of the adjusted number of doctors per 100,000 people across the tertiles for each secondary medical area. In the Q1 group, Nara prefecture had the highest proportion of secondary medical areas. Four of the five secondary medical areas (Nara, Towa, Chuwa, and Nanwa) belonged to Q1. By contrast, in the Q3 group, Kyoto prefecture had the highest proportion of secondary medical areas. Four of the six secondary medical areas (Tango, Chutan, Nantan, and Yamashirominami) belonged to Q3.

Table 3 shows the results of the ordinal logistic regression analysis. A statistically significant association was observed in the crude model when the number of CWFs increased by five or more. Moreover, a significant association was observed in the adjusted model when the number of facilities increased by 10 or more (odds ratio: 5.1, 95% CI: 1.02 to 25.8).

**Table 3:** Crude and adjusted odds ratios of the association between the adjusted number of doctors per 100,000 people and changes in the number of child welfare facilities. *The covariates were medical expenses subsidies for infants, retail sales floor area per population, income per population, and hours of sunlight. CIs: Confidence intervals; Ref: Reference

	Crude				Adjusted*			
Changes in the number of child welfare facilities	Odds ratio	p-value	95% CIs	Brant test	Odds ratio	p-value	95% CIs	Brant test
0 or fewer	Ref			0.73	Ref			0.96
1-4	3.1	0.07	0.9 to 10.6		2.9	0.11	0.8 to 10.4	
5-9	5.8	0.01	1.6 to 21.4		2.6	0.20	0.6 to 11.0	
10 or more	16.6	<0.001	4.3 to 64.9		5.1	0.046	1.02 to 25.8	

Table 4 presents the results of the linear regression analysis. A statistically significant increase in the crude model was observed when the number of CWFs increased by five or more compared with no increase. In the adjusted model, a statistically significant increase was observed when the number of facilities increased by 10 or more (beta coefficient: 54.6, p = 0.01, 95% CI: 13.2 to 96.1).

**Table 4:** Crude and adjusted beta coefficients of the association between the adjusted number of doctors per 100,000 people and changes in the number of child welfare facilities. *The covariates were medical expenses subsidies for infants, retail sales floor area per population, income per population, and hours of sunlight. CIs: Confidence intervals; Ref: Reference

	Crude			Adjusted*		
Changes in the number of child welfare facilities	Coefficient	p-value	95% CIs	Coefficient	p-value	95%CIs
0 or fewer	Ref			Ref		
1-4	19.1	0.29	-16.5 to 54.7	15.8	0.36	-18.6 to 50.2
5-9	38.4	0.046	0.7 to 76.2	14.5	0.46	-24.6 to 53.7
10 or more	85.4	<0.001	49.8 to 121.0	54.6	0.01	13.2 to 96.1

Table 5 presents the results of the linear regression analysis as the sensitivity analysis. In the sensitivity analysis, both the exposure and outcome variables were treated as continuous. A statistically significant positive association was observed in the crude and adjusted models. Additionally, the linear regression analysis was performed using the change in the number of nursery schools as the exposure variable. A significant positive association was observed in the crude model. However, no statistically significant association was found in the adjusted model.

**Table 5:** Crude and adjusted beta coefficients for associations between the adjusted number of doctors per 100,000 people and changes in the numbers of child welfare facilities and nursery schools (all variables continuous). *The covariates were medical expenses subsidies for infants, retail sales floor area per population, income per population, and hours of sunlight. Coefficients were estimated using a linear regression model with restricted cubic splines (four knots at the 5th, 35th, 65th, and 95th percentiles of changes in the number of child welfare facilities and nursery schools). The reference value for changes in the number of child welfare facilities was set at -4. The reference value for changes in the number of nursery schools was set at -3. CI: Confidence interval

	Crude				Adjusted*			
	Coefficient	p-value	95% CI	p for non-linearity	Coefficient	p-value	95% CI	p for non-linearity
Changes in the number of child welfare facilities	1.6	<0.001	1.0 to 2.2	0.32	1.05	0.01	0.3 to 1.8	0.81
Changes in the number of nursery schools	4.8	<0.001	2.7 to 6.8	0.6	2.4	0.07	-0.3 to 5.1	0.79

## Discussion

This study examined the association between the changes in CWFs and the adjusted number of doctors per 100,000 people. An increase in CWFs was associated with an increase in the adjusted number of doctors per 100,000 individuals. Furthermore, when CWFs increased by 10 or more, the adjusted number of doctors per 100,000 people increased by over 50. This study provides preliminary insights into the potential impact of CWFs on the distribution of doctors.

The distribution of doctors is a major social issue in Japan, and various contributing factors have been reported. Previous studies have identified housing environment, the ability to maintain specialist qualifications, and belonging to the ikyoku as influential factors [4-6]. However, research examining CWFs as an associated factor is limited. Meanwhile, overseas studies have reported that well-developed childcare environments, including CWFs, contribute to physician retention [16], but it remains unclear whether these findings can be directly applied to Japan. In plans for securing doctors involving the MHLW, improvements in childcare environments, including CWFs, are explicitly stated as one of the measures to address the distribution of doctors [11]. This is considered to reflect background factors such as the increasing number of female doctors and the issue of long working hours among doctors. As the new plan for securing doctors began in 2024, ongoing monitoring will be necessary to assess its impact on the distribution of doctors in the future.

In this study, an association between the distribution of doctors and the CWFs was observed in the crude analysis; however, the strength of this association decreased after adjusting for covariates. We believe that this is attributable to the income per taxpayer. In areas with higher income levels, municipal finances tend to be more stable, leading to greater investment in social infrastructure such as the CWFs. At the same time, such areas are also likely to provide more favorable living and working environments for doctors. Therefore, the association observed between the distribution of doctors and the CWFs in the crude analysis may reflect income-related regional characteristics.

A potential negative trend is observed between the number of doctors in secondary medical areas and the level of medical expense subsidies for infants. In this study, medical expense subsidies for infants were found to be most generous in Q3 areas. This finding may be influenced by regional characteristics. Secondary medical areas with fewer doctors, such as those in Q3, tend to include a larger proportion of towns and villages, where the pediatric population is relatively small. Consequently, even if medical expense subsidies for infants are enhanced, the financial impact on local government budgets may be limited. In contrast, secondary medical areas with a higher number of doctors, such as those in Q1, tend to include more urban areas and have larger pediatric populations. In such regions, uniformly enhancing medical expense subsidies for infants may impose a substantial fiscal burden, potentially leading to the implementation of certain restrictions. However, Japan operates under a universal health insurance system, which maintains a relatively uniform level of out-of-pocket medical expenses for all citizens. Furthermore, doctors are generally regarded as a high-income professional group; therefore, it is unlikely that differences in medical expense subsidies for infants prevent doctor retention in Q1 areas. Nevertheless, further research is required to examine this issue.

This study has two major strengths. First, it offers novelty by examining the association between the CWFs and the distribution of doctors, which has rarely been investigated in previous research. This contributes new insights to the literature. Second, the study addresses and overcomes the limitations of previous studies by evaluating the distribution of doctors using the adjusted number of doctors per 100,000 people published by the MHLW. By contrast, earlier studies assessed the distribution of doctors based on the number of doctors per 100,000 people [1-3,17-19], which failed to account for local healthcare needs and other critical factors. The adjusted number of doctors per 100,000 people used in this study incorporates various elements such as medical demand and supply [11]. Therefore, by adopting the adjusted number of doctors per 100,000 people as an outcome variable, we could address the limitations of previous studies and conduct a more robust evaluation.

However, this study has some limitations. First, we used Toyo Keizai Inc.’s “Favorable Living Environment: Municipality Ranking 2024” as an indicator of the childcare environment [12]. One variable from each category was selected and used as the covariate. As these variables were selected based on the researcher’s convenience, some bias might have been introduced. However, previous studies have identified these variables as factors that may influence migration [20-23]. Therefore, we considered it appropriate to include these variables as covariates.

Second, we did not assess ecological fallacy and unmeasured confounders, including individual-level factors. Regarding individual-level factors, previous studies have indicated that a doctor’s gender and years of experience are associated with the distribution trends of doctors [24]. This study used Japanese government statistics, which were aggregated and did not include individual-level data. Further research is required to explore the unmeasured confounders and confirm the association between individual-level factors and our results.

Third, in this study, we focused on secondary medical areas in the Kinki and Chugoku regions in order to minimize the influence of differences in medical environments. As a result, the sample size in each group became small, which may have introduced instability in the estimates and random error, leading to wide CIs. However, to our knowledge, this is the first study to examine the association between the CWFs and the distribution of doctors, and previous studies on factors influencing this association are limited. Future studies should include a broader range of regions and investigate potential confounding factors in more detail.

Fourth, in this study, to address potential reverse causality, we used data from 2016 for the exposure variable and data from 2020 for the outcome variable. By shifting the time points, it is considered that the impact of reverse causality could be partially mitigated. However, the distribution of doctors is often influenced by long-term regional trends, which we were unable to fully adjust for in this study.

Finally, this sample was limited to the Kinki and Chugoku regions of Japan. Consequently, since the results of this study are specific to certain regions, it remains unclear whether the findings can be generalized to other regions in Japan. However, the medical environment of the western and eastern regions in Japan differs [25]. Analyzing these different environments may make it difficult to accurately assess the association between the CWFs and the distribution of doctors because of the influence of unmeasured confounders. Therefore, this study focused on the Kinki and Chugoku regions, with relatively similar medical environments, to minimize the impact of unmeasured confounding factors.

## Conclusions

This study examined the association between the CWFs and the distribution of doctors, using open data published by the Japanese government ministries. The secondary medical areas where the CWFs significantly increased were classified into the group with the highest adjusted number of doctors per 100,000 people. These findings suggest that there may be an association between increasing CWFs and the adjusted number of doctors per 100,000 people.

## Appendices

Appendix 1: Scoring system for medical expenses subsidy for infants

Age Eligibility for Medical Expense Assistance Outpatient and Hospitalization (Point)

 Preschool: 1

 Under 10 years old: 2

 Under 13 years old: 3

 Under 16 years old: 4

 Under 19 years old: 5

Income Limit (Point)

 Presence: 0

 Absence: 1

Partial Out-of-Pocket Payment (Point)

 Presence: 0

 Absence: 1

Scoring Range 2 to 12
